# Characterization of respiratory compromise and the potential clinical utility of capnography in the post-anesthesia care unit: a blinded observational trial

**DOI:** 10.1007/s10877-019-00333-9

**Published:** 2019-06-07

**Authors:** Frances Chung, Jean Wong, Michael L. Mestek, Kathleen H. Niebel, Peter Lichtenthal

**Affiliations:** 1 Department of Anesthesia and Pain Management, University Heath Network, University of Toronto, 399 Bathurst Street, McL2-405, Toronto, ON M5T 2S8 Canada; 2 Medtronic Minimally Invasive Therapies Group, Boulder, CO USA; 3 Department of Anesthesiology, University of Arizona Medical Center, Tucson, AZ USA

**Keywords:** Capnography, IPI, PACU, Perioperative care, Respiratory compromise, Respiratory monitoring

## Abstract

The utility of capnography to detect early respiratory compromise in surgical patients after anesthesia is unclear due to limited prospective data. The purpose of this trial was to determine the frequency and duration of capnography-detected respiratory adverse events in the post-anesthesia care unit (PACU). In this prospective observational trial, 250 consenting patients undergoing elective surgery with general anesthesia were monitored by standard monitoring together with blinded capnography and pulse oximetry monitoring. The capnography notification settings were adjusted to match nursing (Level II) and physician (Level I) alarm thresholds. 163 (95%) patients had a Level II notification and 135 (78%) had a Level I notification during standard monitoring. The most common events detected by the capnography monitor included hypocapnia, apnea, tachypnea, bradypnea and hypoxemia, with silent notification duration for these events ranging from 17 ± 13 to 189 ± 127 s. During standard monitoring, 15 respiratory adverse events were reported, with 8 events occurring when valid blinded/silenced capnography and pulse oximetry data was collected simultaneously. Capnography and the Integrated Pulmonary Index™ algorithm (IPI) detected respiratory adverse events earlier than standard monitoring in 75% and 88% of cases, respectively, with an average early warning time of 8 ± 11 min. Three patients’ blinded capnography was unblinded to facilitate clinical care. Respiratory adverse events are frequent in the PACU, and the addition of capnography and IPI to current standard monitoring provides potentially clinically relevant information on respiratory status, including early warning of some respiratory adverse events.

*Trial registration* ClinialTrials.gov Identifier NCT02707003 (https://clinicaltrials.gov/ct2/show/NCT02707003).

**Electronic supplementary material:**

The online version of this article (10.1007/s10877-019-00333-9) contains supplementary material, which is available to authorized users.

## Introduction

Postoperative respiratory compromise, often identified as hypoxemia, is common, and can be severe and prolonged [1, 2]. Most postoperative respiratory complications occur within the first 12–24 h after surgery in the post-anesthetia care unit (PACU) and ward settings [3–5]. Patients who suffer such respiratory adverse events during immediate recovery are at increased risk for further respiratory complications following their PACU stay [6, 7]. Early identification of at-risk patients prior to PACU discharge may allow these patients to be directed to a higher level of care or be given increased vigilance in lower acuity settings, such as the general care floor [5, 8].

Lee et al. reported that 97% of the closed claims of postoperative respiratory depression probably could have been prevented by better monitoring [9]. For patients receiving opioid analgesia medication, combined oxygenation and ventilation monitoring has been recommended by several governing bodies, such as the American Society of Anesthesiologists (ASA), Joint Commission and Anesthesia Patient Safety Foundation [10–14].

While pulse oximetry for oxygenation monitoring is part of standard monitoring in the PACU, hypoventilation cannot reliably be detected by pulse oximetry when patients are on supplemental oxygen [15–17]. Combined with pulse oximetry, capnography, which measures end-tidal CO_2_ (EtCO_2_, also called PetCO_2_), pulse rate (PR), and respiration rate (RR), can provide a more complete characterization of pulmonary function. In addition, the Integrated Pulmonary Index™ algorithm (IPI), which is an algorithm-derived value based on SpO_2_, EtCO_2_, PR and RR, can provide an index of patient ventilatory status [18]. Despite the potential utility for ventilation monitoring in identifying patients at risk for postoperative respiratory adverse events, it is often not used in tandem with oxygenation monitoring [19, 20]. Thus far, oxygenation and ventilation patterns of surgical patients have not been well-characterized in the PACU, limiting what is known about the potential clinical utility of combined pulse oximetry and capnography monitoring in the post-operative setting.

The primary objective of this pilot trial was to determine the frequency and duration of respiratory adverse events such as hypercapnia, hypocapnia, hypoxemia, apnea, and upper airway obstruction in the PACU, identified by blinded ventilation monitoring during standard monitoring. A secondary trial objective was to determine if capnography may provide ventilation information for earlier detection and intervention than standard monitoring. This secondary objective included assessment of the performance of IPI and the Apnea-SAT Alert algorithm in detecting respiratory adverse events. In addition to these trial objectives, we performed post hoc analysis to explore whether use of IPI has the potential to reduce the number of notifications to the bedside provider, compared to individual capnography and oximetry parameter alerts, when respiratory adverse events occur. These preliminary data will be used to characterize respiratory compromise in the PACU and provide a basis for future interventional studies powered to determine the efficacy of respiratory monitoring to reduce the frequency of critical respiratory adverse events in the immediate postoperative setting.

## Methods

### Trial design and participants

This prospective multi-center observational pilot trial was conducted at Toronto Western Hospital and University of Arizona Medical Center after Institutional Review Board (IRB) approval. Patient enrollment and consent began in February 2016 and the last patient follow-up was completed in June 2017 (ClinicalTrials.gov Identifier: NCT027070030, date of registration 7 March 2016). Patients enrolled before clinical trial registration were excluded from data analysis. This manuscript adheres to the Strengthening the Reporting of Observational Studies in Epidemiology (STROBE) statement.

Patient inclusion criteria were: (1) adult ≥ 18 years, (2) ASA score II–IV, (3) patients scheduled for an elective surgery requiring general anesthesia, (4) duration of general anesthesia > 1.5 h, (5) requirement of intraoperative opioids, (6) PACU stay ≥ 45 min, and (7) expected to be transferred from the PACU to an in-patient setting. Exclusion criteria were: (1) ambulatory surgery, (2) physical inability to wear oral/nasal capnography sampling filterline or finger sensors, or (3) pregnancy. Supplemental oxygen was administered to patients as per usual standard clinical practice at the participating institutions.

### Trial procedure

Upon transfer from the operating room (OR) to the PACU, fulfillment of inclusion and exclusion criteria was confirmed, and patients who did not meet the criteria were withdrawn from the trial. In addition to standard monitors (Table 1), all patients were monitored using a Capnostream™ 20p (CS20p) monitor, connected to a Nellcor™ Max A disposable finger pulse oximeter sensor and a Microstream™ Smart CapnoLine™ Plus O_2_ sampling line (Medtronic, Inc., Boulder, CO) to sample oral and nasal CO_2_. The Capnostream™ monitor screen was blinded and all alerts were silenced. The second pulse oximeter (an addition to standard pulse oximetry monitoring) was used for data collection and IPI and Apnea-SAT Alert algorithm calculations.

**Table 1:** Comparison of site-specific standard monitoring alert settings to the blinded and silenced capnography alert settings used during the trial to define respiratory adverse events

Monitored respiratory adverse event	Site-specific standard monitoring alert settings		Capnostream™ monitor default notification settings (no alert delay)	Capnography alert settings used during trial	
	Site 1	Site 2		Level II event (nurse notification)	Level I event (physician notification)
Tachypnea	≥ 30 bpm, no delay	≥ 60 bpm	≥ 30 bpm	≥ 25 bpm for more than 15 s	≥ 30 bpm for more than 30 s
Bradypnea	≤ 8 bpm, no delay	≤ 4 bpm	≤ 5 bpm	≤ 8 bpm for more than 15 s	≤ 6 bpm for more than 30 s
Hypercapnia	N/A	N/A	≥ 60 mmHg	≥ 55 mmHg for more than 15 s	≥ 60 mmHg for more than 30 s
Hypocapnia	N/A	N/A	≤ 15 mmHg	≤ 25 mmHg for more than 15 s	≤ 25 mmHg for more than 30 s
Tachycardia	≥ 130/min, no delay	160/min, no delay	≥ 140/min	≥ 120/min for 15 s	≥ 120/min for 30 s
Bradycardia	≤ 50/min, no delay	≤ 40/min, no delay	≤ 50/min	≤ 40/min for 15 s	≤ 40/min for 30 s
Hypoxemia	≤ 90%, no delay	≤ 90%, no delay	≤ 85%	≤ 90% for more than 15 s	≤ 90% for more than 30 s
Apnea	≥ 20 s	N/A	≥ 30 s	≥ 10 s in a 15 min epoch	≥ 10 s twice in a 15 min epoch

*bpm* breaths per minute

The IPI is an algorithm-derived parameter based on EtCO_2_, RR, SpO_2_, and PR designed to provide an uncomplicated, inclusive assessment of patient ventilatory status. The IPI value, updated every 1 s, is calculated using the average of the last 15 s of each parameter. It is displayed as a single indexed value from 1 to 10, where 8–10 indicates the patient is within a normal range, 5–7 indicates that the patient may need attention, and 1–4 indicates a need for intervention [18]. In this trial, the composite IPI derived from capnography and pulse oximetry was evaluated at IPI value 3, which indicates that the patient requires intervention [18]. The Apnea-SAT Alert algorithm reports the hourly rate of apnea events > 10 s. Patient monitoring data and alerts were wirelessly transmitted to a clinical observation tool on a tablet, allowing the trial coordinator to view the silenced CS20p alerts, verify sensor placement, and track clinical interventions as needed. The clinical observation tool was also used as a mobile case report form to monitor and record medical staff interventions in response to alarm notifications and observed events, including standard monitoring (ECG, impedance RR, pulse oximetry, and blood pressure). Monitoring continued for a minimum of 45 min until patients were transferred out of the PACU and chart review continued for 24 h after patient transfer to allow for tracking of post-PACU adverse events.

### Statistical analysis

There are no previously published studies that utilize capnography to detect respiratory adverse events in the PACU. Therefore, a priori sample size calculation was not possible for this non-powered pilot trial, and we estimated that enrolling 250 patients total, with 125 patients at each site, would be adequate to measure the frequency and duration of PACU respiratory adverse events and critical respiratory adverse events. A respiratory adverse event was defined as being outside normal ranges for oxygenation, ventilation, or both physiological parameters (Table 1) [21, 22], while critical respiratory adverse events were defined as any unanticipated respiratory adverse event requiring active intervention. Interventions included, but were not limited to airway protective methods, opioid or muscle relaxant reversal/antagonism, and airway manipulation.

Since capnography is not standard in the PACU, alarm setting recommendations for respiratory parameters are not widely accepted [21, 22]. Monitor-detected respiratory adverse events were predefined using literature [7] and consensus from clinical experts who utilize institutional-level monitoring standards (FC and PL, Table 1). Levels I and II notifications were based on patient monitor thresholds deemed clinically important enough to notify a physician (Level I) or a nurse (Level II) (Table 1) [7]. Notification settings were decreased for apnea from the device default > 30 s episode notification to match detection of apnea lasting ≥ 10 s [7]. The delay for transmission to the clinical observation tool were set to match Level II notifications, with IPI notification at value 3, which indicates that the patient requires intervention [18].

All data analysis were performed using SAS, version 9.4 (SAS Institute, Inc., Cary, NC, US). Demographic and clinical characteristics were analyzed using descriptive statistics, including counts and percentages for categorical variables and the mean, standard deviation, and range for continuous variables. To determine the frequency and duration of respiratory adverse events identified by capnography, the proportion of patients who experienced respiratory adverse events (Table 1) was determined. Baseline characteristics, along with primary and secondary endpoints, were determined using the cohort of patients who underwent ≥ 45 min monitoring in the PACU and had a 24 h post-PACU chart review. Patients with poor quality device data, defined as having less than 90% of data continuously updated every second, were excluded from the analysis.

Post hoc analysis was performed to examine whether use of IPI can reduce the number of notifications to the bedside provider, compared to the total number of notifications from all individual capnography and oximetry parameters. Briefly, the number of IPI notifications was retrospectively determined for all patients included in trial analysis. IPI notification analysis included cutoffs for IPI values 3 and 2, with either 10 or 30 s delays for each capnography and oximetry parameter alert individually. The total number of single parameter alerts, including SpO_2_, EtCO_2_, RR, PR, and apnea (all parameters), was also determined using 10 and 30 s delays for each capnography and oximetry parameter alert. The sum of alerts from all parameters counted multiple simultaneous individual parameter alerts (e.g., EtCO_2_ and RR) as one notification. Statistical significance was determined using a one-way ANOVA.

## Results

### Demographics, clinical characteristics, and dispositions of patients

**Fig. 1:**
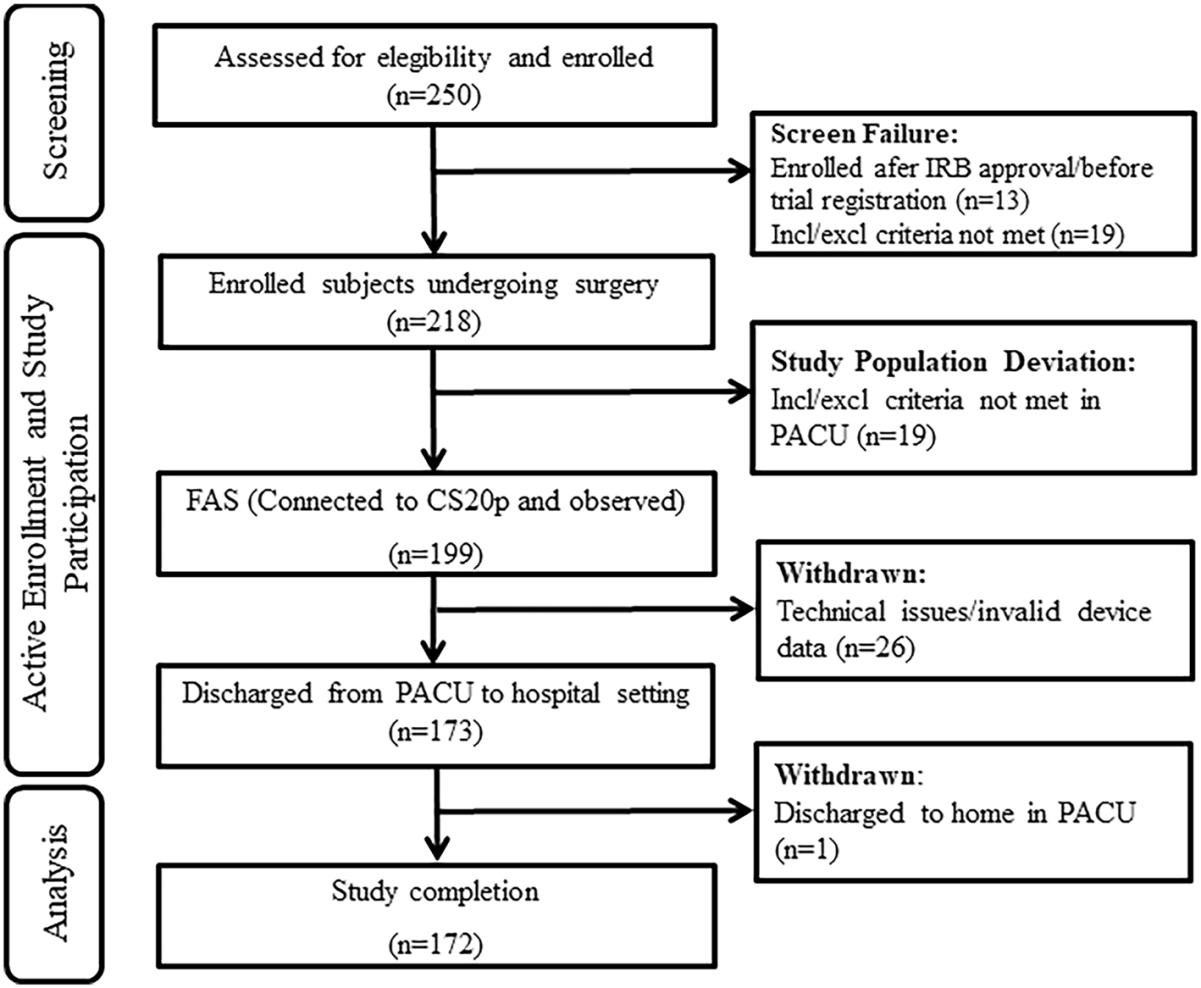
Flow diagram of patient disposition. The full analysis set (FAS) included all patients who were enrolled in the trial, transferred to PACU, and monitored by capnography

A total of 250 patients were enrolled at two trial sites. Thirteen patients at one site were excluded from analysis due to their enrollment after IRB approval and before trial registration with http://clinicaltrials.gov was completed. Upon arrival in the PACU, 19 patients were excluded due to inclusion criteria not being met or trial staff not being available (Fig. 1). Technical issues related to data collection or invalid device data caused the withdrawal of 26 patients and 1 patient was excluded due to being discharged home directly from the PACU. In total, 172 patients completed the trial.

The mean patient age was 53 years, with 40% male participants and an average BMI 33 ± 11 kg/m^2^ (Table 2). The majority of patients were ASA status III. Twenty-nine percent of patients had a history of obstructive sleep apnea (OSA), and a majority of patients with OSA used continuous positive airway pressure (CPAP) at home. The average duration of anesthesia was 215 ± 117 min. The average supplemental O_2_ rate in the PACU was 3.8 ± 2.4 L/min, delivered to 81 patients by mask and 82 patients by nasal cannula; 82% of patients were transported out of the PACU with supplemental O_2_. Eighty-six percent of patients were transferred to the surgical ward and 14% had planned ICU or intermediate care admission, with 6% requiring non-invasive positive pressure ventilation (Table 2). The 24 h post-PACU follow-up indicated that 22% were connected to intravenous patient-controlled analgesia (PCA). No rapid response or codes occurred in the PACU or during the 24 h post-PACU period.

**Table 2:** Demographics and clinical characteristics of 172 patients who completed the trial

Demographic or clinical characteristic	Mean ± SD or mean N (%)^a^
Age (years)	53 ± 15
Gender	
Male, female	69 (40), 103 (60)
BMI (kg/m^2^)	33 ± 11
Race	
White	147 (85.5)
Black or African American	11 (6.4)
Asian	7 (4.1)
Other	7 (4.1)
Pre-op ASA score	
II, III, IV	62 (36), 103 (60), 7 (4)
Medical history	
Cardiovascular	106 (61.6)
Respiratory	99 (57.6)
OSA	50 (29.1)
Patient was on non-invasive pressure at night	34 (19.8)
COPD	6 (3.5)
Asthma	34 (19.8)
Other pulmonary/respiratory disease	44 (25.6)
Gastrointestinal	93 (54.1)
Genitourinary	22 (12.8)
Endocrine	64 (37.2)
Musculoskeletal	101 (58.7)
Hematological	48 (27.9)
Immunological	39 (22.7)
Surgery type	
Abdominal [% laparoscopic]	69 (40.1) [81.2]
Craniotomy	17 (9.9)
ENT	6 (3.5)
Gynecology [% laparoscopic]	10 (5.8) [20.0]
Orthopedic [% spine]	58 (33.7) [86.2]
Plastic	1 (0.6)
Thoracic [% thoracoscopic]	4 (2.3) [25.0]
Urology	4 (2.3)
Vascular	3 (1.7)
Anesthesia duration (min)	215 ± 117
Length of stay in PACU (min)	150 ± 76
Monitoring duration (min)	84 ± 31
Patient connected to PCA in PACU for transport	38 (22)
Daily morphine milligrams equivalents in PACU	7.2 ± 9.2
Supplemental oxygen (O_2_) flow rate (L/min)	
Operating room [n/N, % patients]	7.9 ± 1.3 [168/170, 98]
PACU [n/N, % patients]	3.8 ± 2.4 [172/172, 100]
Post PACU [n/N, % patients]	2.9 ± 1.7 [141/172, 82]
Supplemental O_2_ delivery by mask (n/N, %)	
Operating room (post-extubation, transfer to PACU)	165/168, 98
PACU	81/172, 48
Post-PACU (on transfer/discharge from PACU)	4/141, 3
Supplemental O_2_ delivery by nasal cannula (n/N, %)	
Operating room (post-extubation, transfer to PACU)	2/168, 1
PACU	82/172, 48
Post-PACU (on transfer/discharge from PACU)	132/141. 94
Transport out of PACU on oxygen	142 (82.6)
Noninvasive positive pressure after PACU	11 (6.4)
Transfer out of PACU to	
Surgical ward	148 (86)
Intermediate care floor	1 (0.6)
Planned intensive care unit	22 (12.8)
Other	1 (0.6)

*BMI* body mass index, *OSA* obstructive sleep apnea, *COPD* chronic obstructive pulmonary disease, *PACU* post-anesthesia care unit, *PCA* patient-controlled analgesia

^a^Mean ± SD or N (%) where appropriate

### Respiratory adverse events identified by blinded capnography

Of the 172 patients who completed the trial, 163 (95%) had ≥ 1 Level II (nurse) notification, and 135 (78%) had ≥ 1 Level I (physician) notification. The most common respiratory adverse events detected by capnography included hypocapnia, apnea, tachypnea, bradypnea and hypoxemia, with notification duration ranging from 17 ± 13 to 189 ± 127 s (Table 3). About 4% (N = 280) of notifications were recognized by the trial coordinator as invalid, defined by poor sensor placement, and were removed from the analysis. Within Level I notifications, 2 patients had hypercapnia, 48 had hypocapnia, 113 had apnea, 6 had tachypnea, and 53 had bradypnea.

**Table 3:** Frequency, duration, and rate of monitored respiratory adverse events identified by capnography

Event type	Patients with event N (%)	Total events (n)	Notification duration (s) mean ± SD	Rate (total events/min)	Rate 95% CI
Hypercapnia Level I	2 (1.16)	4 (0.1)	71.3 ± 19.5	0.03	0.01–0.07
Hypercapnia Level II	15 (8.72)	166 (7.0)	31.6 ± 27.6	1.15	0.99–1.34
Hypocapnia Level I	48 (27.91)	295 (7.6)	94.7 ± 101.3	2.05	1.83–2.30
Hypocapnia Level II	66 (38.37)	421 (17.7)	25.4 ± 7.1	2.93	2.66–3.22
Apnea Level I (one ≥ 10 s alert in 15 min epoch)	113 (65.70)	2953 (76.3)	19.1 ± 13.9	20.53	19.80–21.28
Apnea Level II (more than one ≥ 10 s alert in 15 min epoch)	45 (26.16)	54 (2.3)	16.9 ± 13.4	0.38	0.29–0.49
Tachypnea Level I	6 (3.49)	24 (0.6)	189.3 ± 127.2	0.17	0.11–0.25
Tachypnea Level II	65 (37.79)	505 (21.3)	38.9 ± 39.9	3.51	3.22–3.83
Bradypnea Level I	53 (30.81)	359 (9.3)	88.6 ± 57.6	2.50	2.25–2.77
Bradypnea Level II	98 (56.98)	809 (34.1)	28.7 ± 15.7	5.62	5.25–6.03
Hypoxemia Level I	36 (20.93)	160 (4.1)	112.9 ± 186.9	1.11	0.95–1.30
Hypoxemia Level II	56 (32.56)	300 (12.6)	21.2 ± 4.3	2.09	1.86–2.34
Tachycardia Level I	16 (9.3)	77 (2.0)	140.2 ± 139.9	0.54	0.43–0.67
Tachycardia Level II	12 (6.98)	41 (1.7)	22.3 ± 4.9	0.29	0.21–0.39

During monitoring in the PACU, 172 patients experienced a total of 3872 Level I (physician) notifications and 2373 Level II (nurse) notifications, resulting in 6245 total respiratory adverse events. The combined monitoring time for 172 patients was 14,384 min

In total, 47 adverse events were reported by standard monitoring between the end of surgery and completion of the 24 h follow-up, including 24 respiratory adverse events (Table 4). The other 23 adverse events were mainly hypertensive or hypotensive episodes. Throughout the trial, 16 (9.25%) patients were observed by standard monitoring to have decreased oxygen saturation. In the PACU, standard monitoring detected 15 respiratory adverse events, including 1 case (0.58%) of decreased respiratory rate and 1 case (0.58%) of hypercapnia in the PACU (Table 4).

**Table 4:** Summary of adverse events observed during trial

Adverse event	Number of patients with adverse event, detected by standard monitoring	Frequency of adverse event (%)
Oxygen saturation decreased	15	8.67
Oxygen saturation decrease post PACU	1	0.58
Increased or high heart rate	1	0.58
Respiration rate decrease	2	1.16
Labored breathing with CPAP PACU	1	0.58
Drowsiness requiring Narcan post-PACU	1	0.58
Drowsiness in OR requiring Narcan	1	0.58
Mild airway obstruction (CPAP)	2	1.16
Blood pressure dropped	1	0.58
Blood pressure reading high	8	4.62
Transient blood pressure increase	1	0.58
Blood pressure fluctuation	4	2.3
Hypotensive	4	2.3
Hypertensive	4	2.3
Hypercapnia	1	0.58
Summary of adverse events (N = 173)		
Total adverse events	47	27.2
Total patients with adverse events	39	22.5
Total patients with respiratory compromise adverse events	22	12.7
Severity of adverse event (N = 47)		
Mild	38	80.9
Moderate	9	19.1
Severe	0	0
Relationship to surgical procedure or recovery (N = 47)		
Not related	5	10.6
Possibly related	31	66
Probably related	11	23.4

### Early detection of respiratory adverse events

Due to some respiratory adverse events occurring before the start or after the end of blinded capnography monitoring, 8 of the 15 PACU events had simultaneous continuous capnography and standard monitoring. Capnography (EtCO_2_, RR, and/or apnea alerts) and IPI detection of standard monitoring-reported respiratory adverse events were earlier in 75% and 88% of cases, respectively, with 3 patients’ respiratory adverse events detected between 16 and 25 min before standard monitoring identified the adverse event (Table 5). Six of the eight respiratory adverse events that occurred during continuous monitoring were low O_2_ saturation detected by capnography and IPI (Online Resources 1A and B). In both capnography and IPI tracing examples, significant fluctuation occurred in capnography and pulse oximetry parameters and IPI value before the respiratory adverse event was reported by standard monitoring (dashed vertical line). The average early warning time for capnography-detected respiratory adverse events was 8.3 ± 11 min. As a non-powered observational trial, the sensitivity and specificity of capnography and pulse oximetry in detecting early signs of respiratory compromise were not determined. In addition, because the average length of monitoring was 84 ± 31 min and the Apnea-SAT Alert algorithm reports apnea and oxygen desaturation events as hourly rates, the performance of this algorithm could not be meaningfully evaluated.

**Table 5:** Early detection of standard monitoring reported respiratory adverse events in the PACU by capnography

Case^a^	Standard monitoring-reported respiratory AE	Supplemental O_2_ before respiratory AE	Supplemental O_2_ after respiratory AE	Capnography detected AE	IPI detected AE (IPI ≤ 3)	Length of early AE detection (min)
1	Low O_2_ saturation	4 L/min, NC	8 L/min, FM		Yes	1
2	Low O_2_ saturation	8 L/min, FM	12 L/min, FM	Low EtCO_2_	Yes	21
3	Low O_2_ saturation	None	3 L/min, NC	Low EtCO_2_	Yes	0
4	Low RR	8 L/min, FM	8 L/min, FM		No^b^	0
5	Low O_2_ saturation	3 L/min, NC	5 L/min, NC	Low RR	Yes	16
6	Low O_2_ saturation	2 L/min, NC	5 L/min, NC	Low EtCO_2_, high RR, low RR	Yes	0
7	Low O_2_ saturation	4 L/min, NC	5 L/min, NC	High EtCO_2_, low EtCO_2_, low RR, apnea	Yes	25
8	Hypercapnia	8 L/min, FM	8 L/min, FM	High EtCO_2_	Yes	3
Summary				6/8 = 75%	7/8 = 88%	8.25 ± 10.6 min

*AE* adverse event, *EtCO*_*2*_ end tidal CO_2_, *RR* respiratory rate, *IPI* Integrated Pulmonary Index™, *NC* nasal cannula, *FM* face mask

^a^Of the 15 reported respiratory adverse events in the PACU by SoC, parallel device data was collected for 8 events due to some events occurring before the start or after the end of capnography monitoring

^b^Although blinded capnography monitoring was occurring during this respiratory adverse event, no single parameter capnography notifications occurred. Due to data collection technical issues, the calculated parameters from the CS20p monitor were not recorded and thus, early detection by IPI could not be determined for this case. The low respiration rate was measured by the impedance respiration rate from the ECG lead during standard monitoring

### Critical respiratory adverse events

Critical respiratory adverse events included opioid reversal in two patients (1 in OR and 1 post-PACU), and neuromuscular antagonist for one patient who had breathing difficulty in transit to the PACU (Table 4). In one case, after opioid reversal was administered post-PACU, nursing staff found an unreported fentanyl patch on the patient. In the PACU, this patient had maintained high SpO_2_ levels, but had varying EtCO_2_ and RR patterns that contributed to the IPI < 3 that was repeatedly observed by continuous capnography (Online Resource 2A).

### Respiratory challenges in perioperative setting

In addition to respiratory adverse events, there were several cases of respiratory challenges detected by standard monitoring, including three patients with respiratory insufficiency requiring high flow supplemental oxygen with unplanned CPAP in the PACU or post-PACU (Online Resource 3). Importantly, two patients who each completed 45 min continuous capnography monitoring in the PACU were thereafter removed from the trial device to allow for unblinded capnography monitoring, per attending RNs’ requests. In addition, one patient developed hypercapnia (> 60 mmHg for > 30 s) in the PACU, and the blinded capnography data was unblinded at the request of the attending nurse to utilize in treating the patient (Online Resource 2B).

### IPI notification setting analysis

Compared to the number of notifications from all individual alerts (SpO_2_, EtCO_2_, RR, PR, and apnea), IPI notifications with a value 2 alert setting, using a 10 s delay on alerts for each parameter, resulted in a reduction in notifications (1683 vs. 1356 notifications, Online Resource 4). Increasing the parameter notification delay to 30 s reduced the total notifications numbers to 585 and 487, for all individual parameters and IPI with a value 2 notification setting, respectively (p < 0.001).

## Discussion

This multicenter pilot trial demonstrates that respiratory adverse events are frequent in the PACU, and the addition of capnography to standard pulse oximetry monitoring provides potentially clinically useful information to help identify and prevent respiratory compromise. We characterized the additive value of ventilatory monitoring for postoperative patients over standard monitoring by collecting blinded continuous pulse oximetry and capnography data. Almost 80% of patients had at least one Level I notification. The most common reasons for the notifications were apnea, bradypnea, hypocapnia, tachypnea, and hypercapnia, all of which reflect potentially clinically relevant early warning signs not provided by pulse oximetry alone and thus are missed when capnography is not used.

Although this trial was not statistically powered to assess the impact of combined oxygenation and ventilation monitoring on clinically reported adverse events, we did document 15 such events, most of which were decreases in oxygen saturation, suggesting that unblinded capnography monitoring could have provided the clinical staff with an early warning to these clinically reported adverse events.

Respiratory episodes based on pulse oximetry alone are known to occur frequently in the PACU. Previous reports suggest that that up to 55% of PACU patients experience at least one episode of hypoxemia [1–4, 23]. These hypoxemia episodes often occur ≥ 30 min into the stay when anesthesia providers are not present [23]. Adding capnography monitoring to other monitors has the potential to alert clinicians to these episodes before they occur, allowing for proactive intervention in the PACU prior to discharge to the ward. In a subset of patients in this trial, six of eight patients who had respiratory adverse events detected by standard care also had respiratory episodes detected by the individual blinded capnography parameters (Table 5). Five of these capnography-detected events were identified before standard care monitors reported the respiratory adverse event, with an average warning time of 8 min, suggesting that capnography can provide early warning of patient ventilatory challenges in the PACU. For some patients, continued monitoring on the ward, where vital signs checks occur every 4–6 h, may reduce the incidence of respiratory adverse events [11].

The potential utility of capnography in the PACU is also supported by three cases in this trial in which capnography monitoring was requested by the attending nurse. In two cases, patients were monitored by the blinded trial device for the full 45 min required to participate in the trial, but after this monitoring period ended, the patients were removed from the trial device and connected to the sites’ capnography monitors. In both cases, unblinded capnography was preferred by the nurse due to the patients’ status. In a third patient, the blinded capnography data was unblinded to the nurse due to development of hypercapnia, with EtCO_2_ > 60 mmHg, while oxygen saturation remained near 90%. These cases provide examples of how, in some patients, clinicians prefer to use capnography in addition to standard monitoring, to allow for enhanced patient monitoring and detection of ventilatory challenges. Such cases are supported by a recent meta-analysis demonstrating that compared to standard nursing care, pulse oximetry is 15 times more likely to detect oxygen desaturation. Importantly, compared to pulse oximetry alone, continuous capnography is six times more likely to detect postoperative respiratory depression [20].

While no serious adverse events occurred during the trial, the earlier warning that capnography could provide has potential clinical application because it may provide a longer window for clinicians to intervene and prevent further respiratory compromise. This concept was recently shown in pediatric PACU patients, where the addition of capnography to standard monitoring resulted in less hypopneic hypoventilation and apnea, with the authors reporting this may have been because of more effective nursing staff interventions [24]. Ultimately, combined pulse oximetry and capnography monitoring could help reduce the clinical and economic consequences of respiratory adverse events.

With respect to the performance of algorithms that account for capnography and oximetry parameters, analysis of the Apnea-SAT Alert algorithm, which calculates an hourly rate of apnea and oxygen desaturation events, was not performed due to the short monitoring length of patients (84 ± 31 min). The IPI algorithm, which averages the last 15 s of each parameter reading and is updated every 1 s, detected almost 90% of the respiratory adverse events detected by standard care. Notably, this detection rate was higher than the rate of detection of individual capnography parameters alone. In one case, IPI detected the respiratory adverse event up to 25 min before detection by standard monitoring. These data suggest combining information from pulse oximetry and capnography (SpO_2_, EtCO_2_, PR and RR) into a rapidly updated single index value has the potential to provide an accurate warning of respiratory adverse events.

Nearly half of the episodes detected by capnography were Levels I and II apnea notifications (Table 3). At trial outset, the apnea notification delay was decreased from device default of 30 to 10 s, to align with detection of OSA, which is a challenge in the postoperative setting and is associated with increased cardiovascular complications when severe OSA goes unrecognized [25, 26]. If this notification delay was changed to the default setting (30 s), it would likely report fewer episodes and could reduce alarm fatigue. This is supported by post hoc analysis examining the number of IPI notifications across all patients when using either 10 or 30 s notification delays. Increasing the notification delay from 10 to 30 s significantly decreased the number of IPI value 3 notifications (Online Resource 4). The same was true for IPI value 2 notifications, which produced the lowest number of notifications of any setting explored. Together, this suggests that increasing the apnea alert delay to 30 s can decrease notification frequency and the potential for alarm fatigue. Similar conclusions have been made by other independent trials utilizing IPI, in which the simplified alarm has been shown to be effective in detecting opioid-induced respiratory depression and non-inferior to multiparameter monitoring, while offering the potential to reduce alarm fatigue [27, 28]. In cases of high risk patients with OSA, the clinician could also set the continuous monitoring notifications to a different preferred notification setting, to ensure proper monitoring while avoiding alarm fatigue.

### Limitations

Device data were intentionally blinded to determine the frequency of respiratory adverse events, so clinical interventions were not based on capnography monitoring. Additionally, although 15 clinically reported adverse events occurred in the PACU, 7 of these occurred either before the trial device was connected to the patient, or after the 45 min capnography monitoring was completed. If use of capnography monitoring were standard in the PACU, it is likely that monitoring would have started earlier upon patient arrival in the PACU and continued until transfer out of the PACU, making it more likely that clinically reported adverse events could be detected earlier. A majority of the excluded patients were removed from analysis either due to failure to continue to meet all PACU inclusion and exclusion criteria or due to technical issues with the Wi-Fi-based device data collection system used for recording trial data. Importantly, although the Wi-Fi issues prevented recording of some of the blinded capnography trial data, this did not interfere with the function of ventilation monitoring. Finally, this was a pilot trial not powered to demonstrate potential capnography monitoring-related changes in patient outcomes, limiting our ability to demonstrate that capnography monitoring could have led to better intervention.

## Conclusions

These data indicate that respiratory adverse events are frequent in the PACU and that the addition of capnography to pulse oximetry monitoring, including utilization of IPI, gives potentially clinically useful information on respiratory status. Further interventional studies are warranted to determine if these early warnings to respiratory adverse events reduce adverse patient outcomes.

## Electronic supplementary material

Below is the link to the electronic supplementary material.

Supplementary material 1 (DOCX 318 kb)
