# Supplementary material for: Characterization of respiratory compromise and the potential clinical utility of capnography in the post-anesthesia care unit: a blinded observational trial
Source: J Clin Monit Comput. 2019 Jun 7;34(3):541–51. doi: 10.1007/s10877-019-00333-9 (PMC7205778; doi:10.1007/s10877-019-00333-9)
Supplement: Supplementary file 1 — Supplementary material 1 (DOCX 318 kb) [file 10877_2019_333_MOESM1_ESM.docx]

**Online Resources**

**Characterization of Respiratory Compromise and the Potential Clinical Utility of Capnography in the Post Anesthesia Care Unit: A Blinded Observational Trial**

*Journal of Clinical Monitoring and Computing*

Frances Chung, MBBS,* Jean Wong, MD,* Michael Mestek, PhD,† Kathleen H. Niebel, BSN,† Peter Lichtenthal, MD‡

**Corresponding author**: Frances Chung, MBBS, Department of Anesthesia and Pain Management, University Heath Network, University of Toronto, 399 Bathurst Street, McL2-405, Toronto, ON, M5T 2S8. Telephone: (416) 603-5118 Email: [frances.chung@uhn.ca](mailto:frances.chung@uhn.ca)

ORCID ID: <http://orcid.org/0000-0001-9576-3606>

**Online Resource 1** Capnography tracing of 2 cases in which respiratory adverse events were detected by continuous capnography monitoring before standard monitoring

**Online Resource 2** Capnography tracing of two cases in which clinical interventions occurred due to a respiratory adverse event or respiratory challenge

**Online Resource 3** Summary of patients with respiratory challenges, including some critical respiratory adverse events, that resulted in medical intervention

**Online Resource 4** Number of notifications when using individual alerts for each capnography and oximetry parameter vs IPI

**Online Resource 1** Capnography tracing of 2 cases in which respiratory adverse events were detected by continuous capnography monitoring before standard monitoring, showing Integrated Pulmonary Index™ algorithm, SpO_2_, PR, EtCO_2_, and RR in A) Case 5, in which the patient experienced clinician-reported low O_2_ saturation. Capnography and IPI both detected the respiratory adverse event up to 16 minutes before standard monitoring detected it. B) Case 7, in which the patient experience clinician-reported low O_2_ saturation. Capnography and IPI both detected the respiratory adverse event up to 25 minutes before standard monitoring detected the event.

**
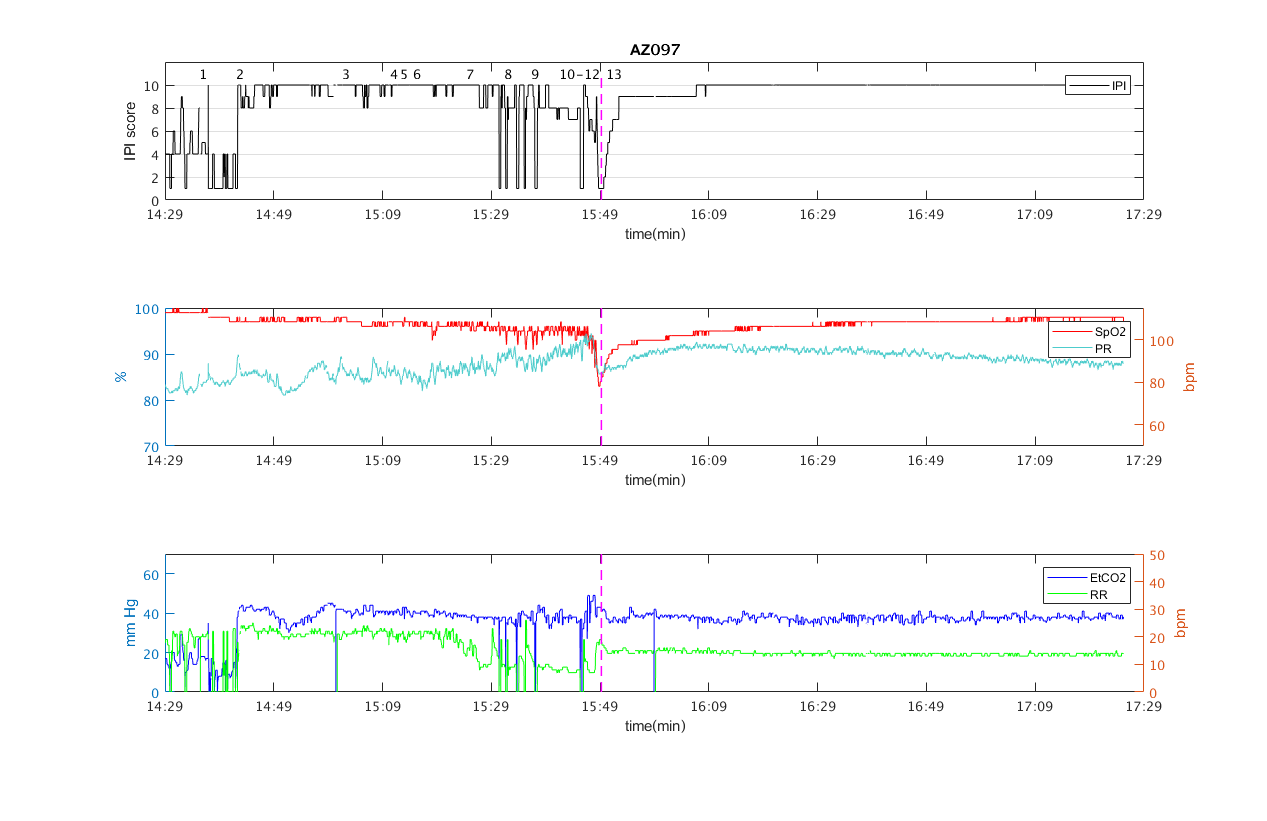

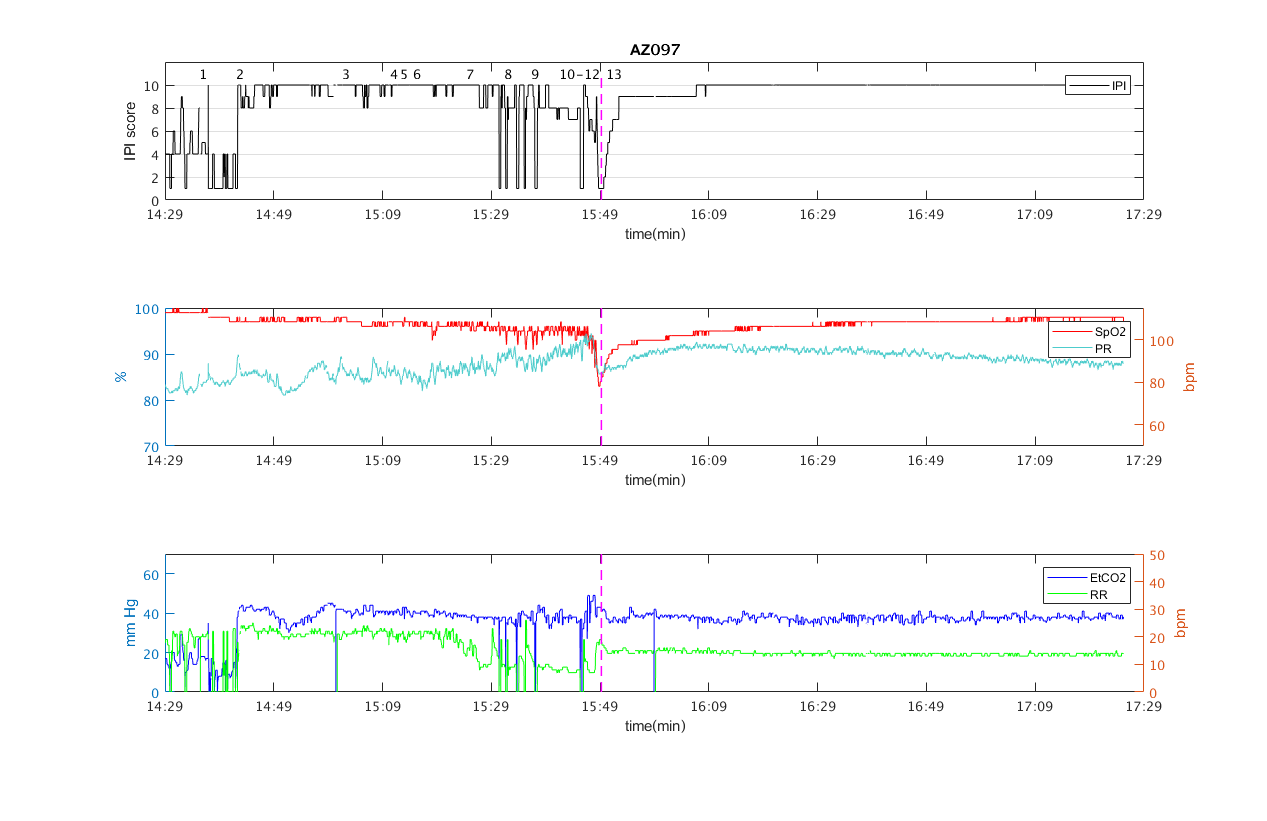
A)**

^1^Nurse: lung sound; ^2^Removed mouth piece; ^3^O_2_ RateChange: 3L/min O_2_ via Microstream™ cannula; ^4 & 5^Disconnected blood pressure cuff for blood draw; ^6^Blood draw; ^7^Placed sensor on right pinky finger; ^8-12^Hydromorphone 0.5mg IV Push (Pain:7 - 9); ^13^O_2_ RateChange: 5L/min O_2_ via Microstream™ cannula. Dashed vertical line represents time of respiratory adverse event.

**
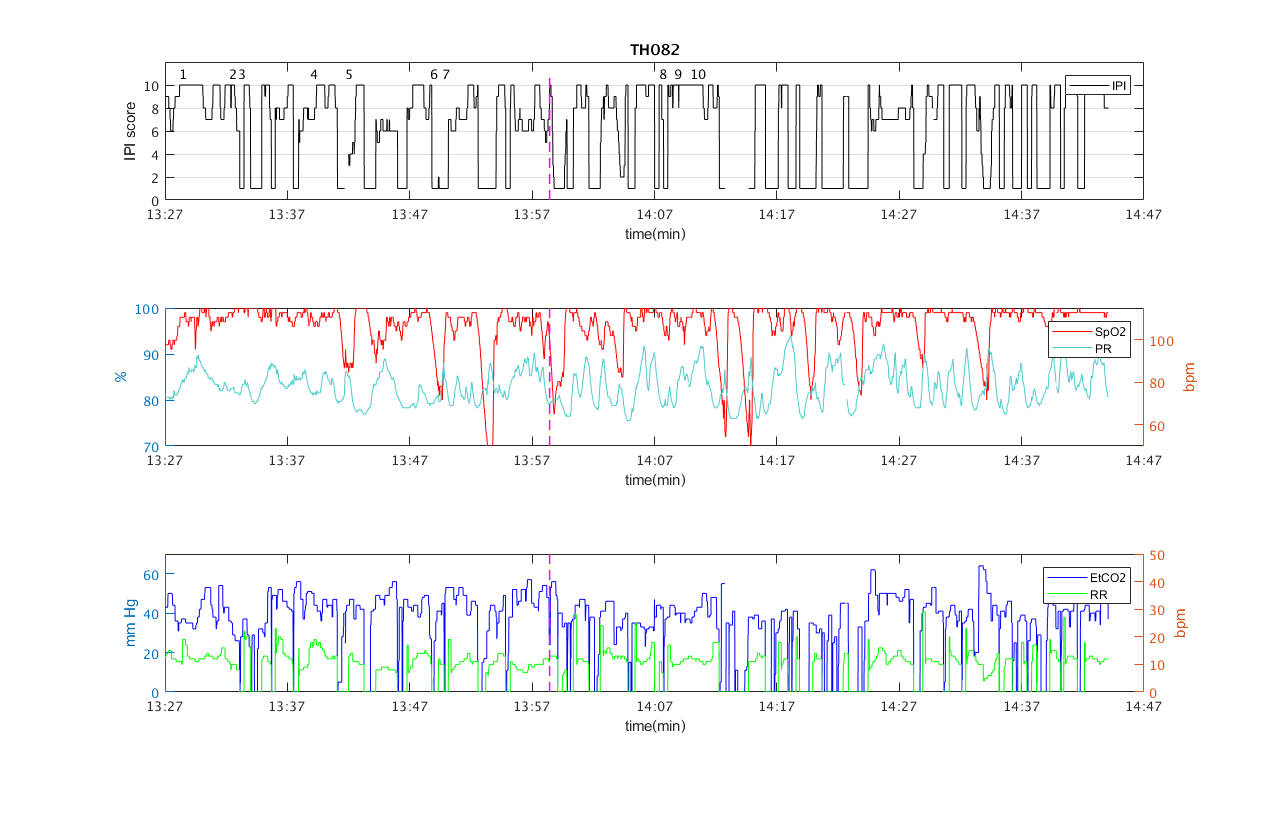

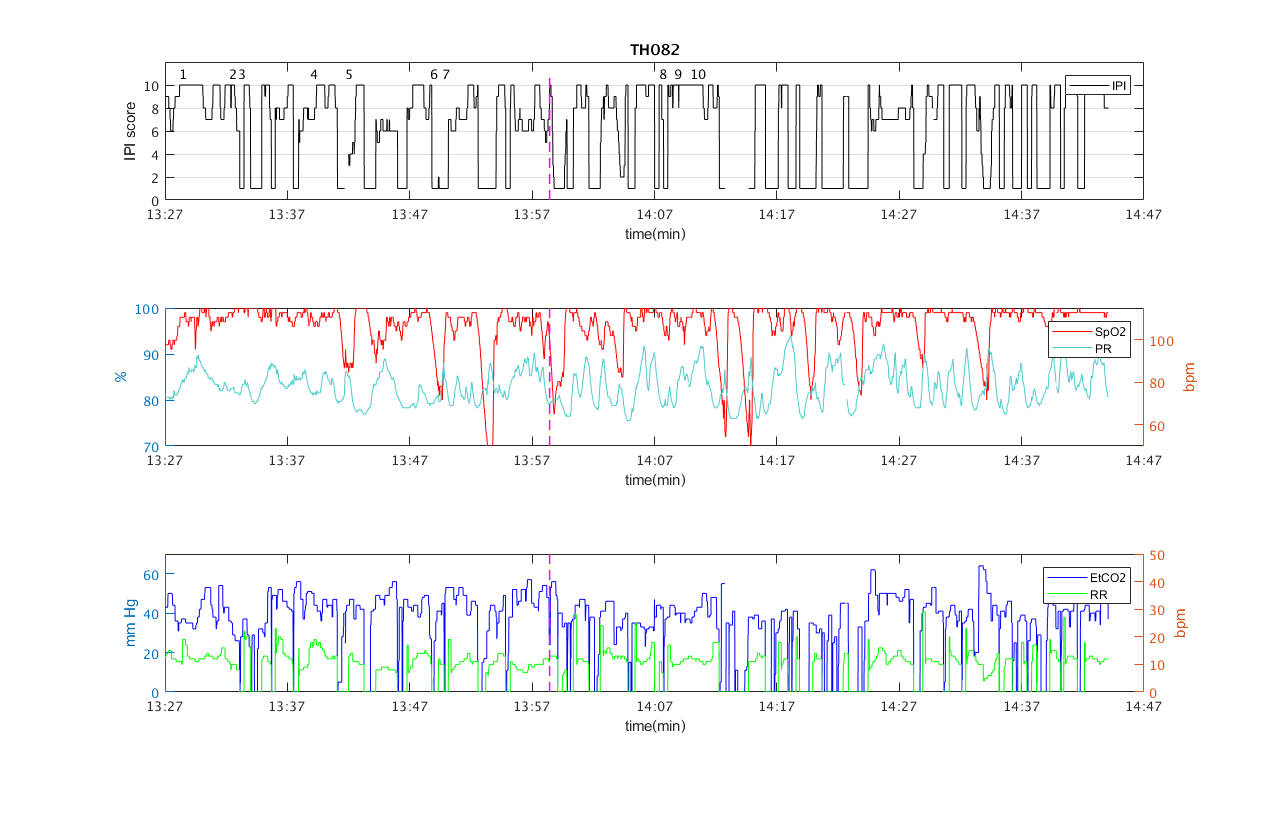
B)**

^1^Assessment +patient talking; ^2, 4, &7^Hydromorphone 5mg IV (Pain:7 - 9); ^3^Fentanyl 25mcg IV Push (Pain:7 - 9); ^5, 6^Fentanyl 50mcg IV Push (Pain:7 - 9); ^8^Change in O_2_: nasal prong to face mask, 6L/min; ^9^Bed 45 degree up + patient talking; ^10^Encouraged coughing. Dashed vertical line represents time of respiratory adverse event.

**Online Resource 2** Capnography tracing of two cases in which clinical interventions occurred due to a respiratory adverse event or respiratory challenge, showing Integrated Pulmonary Index™ algorithm, SpO2, PR, EtCO2, and RR. A) Patient experienced post-PACU drowsiness requiring opioid reversal. B) Case 8, in which the patient developed hypercapnia. This was detected by capnography and IPI up to 3 minutes before standard monitoring detected the respiratory adverse event, and the capnography blind was broken in order to allow the attending nurse to intervene.

##
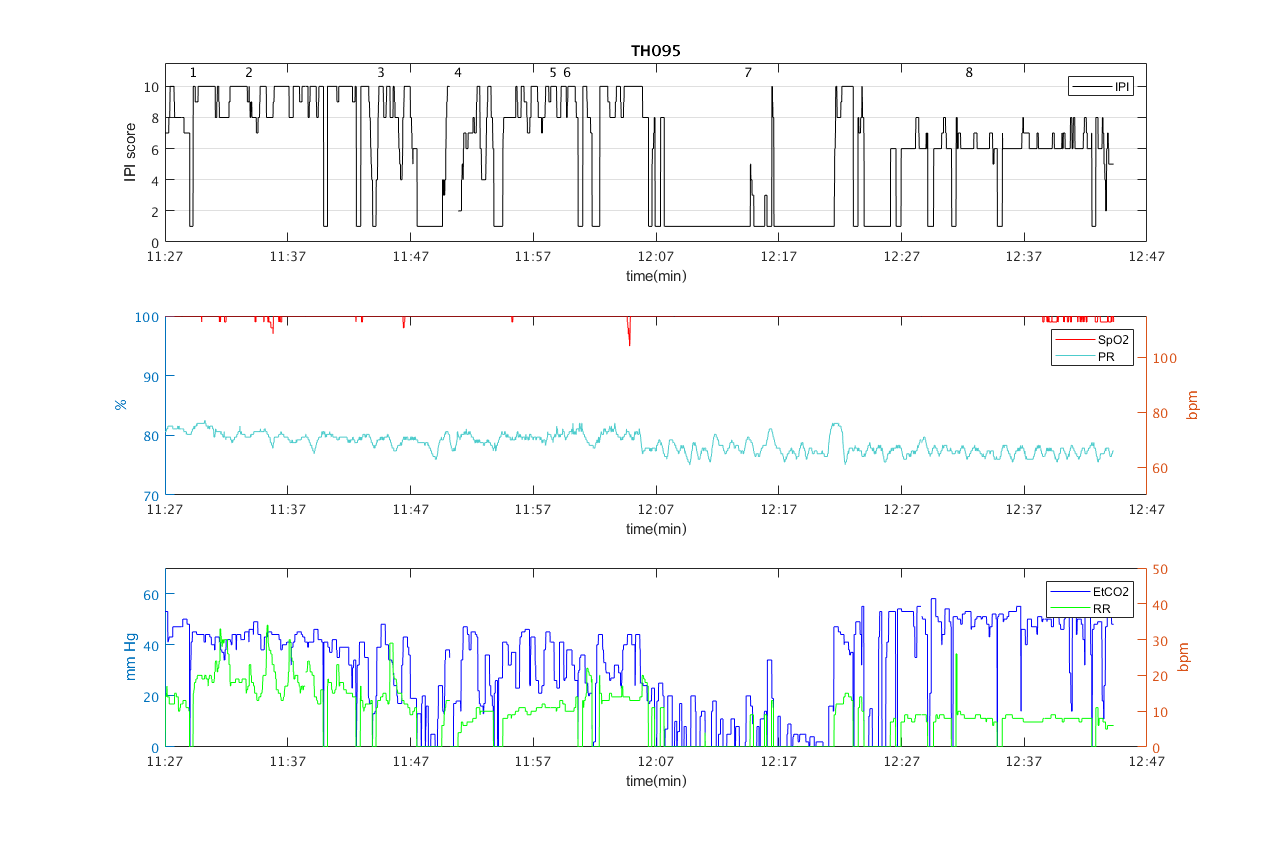
A)

1: Resting; 2: Neurological & nurse assessment; 3: Fentanyl 50mcg IV Push (Pain:4 - 6); 4: Antiemetic medication+ Fentanyl 50mcg IV Push (Pain:4 - 6); 5: Hydromorphone 1mg IV Push (Pain:4 - 6); 6: O_2_ flow change: face mask to nasal prong, 4L/min; 7: Fentanyl 25mg (Pain:4 - 6); 8: Sleeping

## B)

##
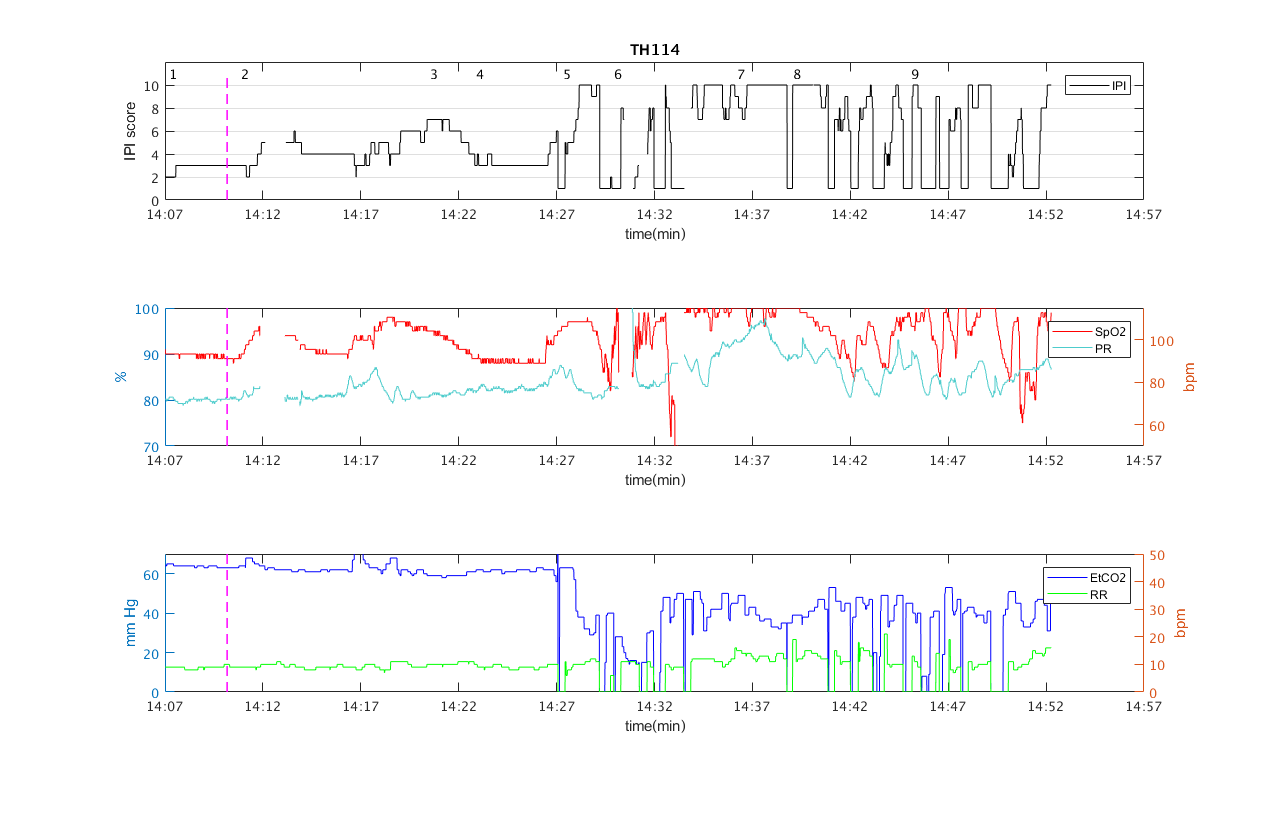


1: PACU nurse informed of high EtCO_2_; 2: PACU nurse informed of SpO_2_ value; 3-5: Sleeping and snoring heavily; 6: Hydromorphone 0.2mg IV (patient controlled analgesia); 7: Sensitivity test+ nurse testing patient's movement; 8: O_2_ Rate Change:4L/min O_2_ via Microstream™ cannula; 9: Antiemetic Medication. Dashed vertical line represents time of respiratory adverse event.

**Online Resource 3** Summary of patients with respiratory challenges, including some critical respiratory adverse events, that resulted in medical intervention.

| **Description of Patient Respiratory Challenge** | **Location** | **Intervention** | **Number of Patients** |
| --- | --- | --- | --- |
| Difficulty breathing | In transit to PACU | Neuromuscular antagonist | 1 |
| Mild Airway Obstruction | PACU | CPAP | 1 |
| Labored Breathing in PACU | PACU | CPAP | 1 |
| Nurse Concern over Ventilation/Oxygenation Insufficiency | PACU | Removed from trial device after 45 min, to allow for unblinded capnography monitoring | 2 |
| Mild Airway Obstruction | Post-PACU | CPAP | 1 |

**Online Resource 4** Number of notifications when using individual alerts for each capnography and oximetry parameter vs IPI (value 3 or 2). For each notification type, a delay of 10 seconds was applied to all capnography and oximetry parameters. The analysis was also performed using a 30 second delay for all capnography and oximetry parameters.

Statistical analysis: 1-way ANOVA, p<0.001; Bonferroni post-hoc analysis: All 10 second delay notification types (All parameters, IPI Value 3, and IPI Value 2) were significantly different than all 30 second delay notification types (p<0.01).
